# Ex vivo study of bacterial coronal leakage in indirect pulp treatment

**DOI:** 10.4317/medoral.18425

**Published:** 2012-12-10

**Authors:** Matilde Ruiz, Pilar Baca, Maria M. Pardo-Ridao, Maria T. Arias-Moliz, Carmen M. Ferrer-Luque

**Affiliations:** 1 DDS, PhD: Assistant Professor, Department of Paediatric Dentistry, School of Dentistry. University of Granada, Campus de Cartuja, Colegio Máximo s/n, Granada, Spain; 2 DDS, MD, PhD: Professor, Department of Preventive Dentistry, School of Dentistry.University of Granada, Campus de Cartuja, Colegio Máximo s/n, Granada, Spain; 3 DDS, Postgraduate Student. University of Granada, Campus de Cartuja, Colegio Máximo s/n, Granada, Spain; 4 DDS, PhD: Assistant Professor, Department of Microbiology, School of Dentistry.University of Granada, Campus de Cartuja, Colegio Máximo s/n, Granada, Spain; 5 DDS, MD, PhD: Associate professor, Department of Dental Pathology and Therapeutics, School of Dentistry. University of Granada, Campus de Cartuja, Colegio Máximo s/n, Granada, Spain

## Abstract

Objective: The aim of this study was to evaluate, ex vivo, bacterial coronal leakage with different antimicrobial agents applied to the dentine for indirect pulp treatment (IPT). Study Design: Sixty extracted teeth were prepared and randomly distributed into 5 groups (n=10): Group 1: no antimicrobial dentine treatment; group 2: 1% chlorhexidine (CHX)+1% thymol varnish (Cervitec®); group 3: 2 % CHX solution; group 4: 40% CHX varnish (EC40™) and group 5: Clearfil™ Protect Bond (CPB). Ten teeth served as controls. The teeth were restored using a resin-modified glass ionomer cement (GIC) and then mounted in a two-chamber device. The coronal access was exposed to Streptococcus mutans for 45 days. The appearance of turbidity in the BHI broth of the lower chamber was considered as specimen leakage. Results: Survival analysis, determined by non parametric Kaplan-Meier and log-rank tests, showed that the best results were for groups EC40™+GIC and GIC alone; yet there were not statistically significant differences between them. All specimens of CPB+GIC and 2% CHX+GIC, leaked at 45 days. Conclusions: In IPT the use of GIC without pretreatment of the dentine and pretreatment with 40% CHX varnish resulted in a significant delay of bacterial coronal leakage.

** Key words:**Streptococcus mutans, bacterial leakage, resin-modified glass ionomer cement, indirect pulp treatment.

## Introduction 

The main objective of the management of the primary and young permanent dentition is to preserve the teeth´s vitality, while causing as little trauma as possible to the pulp (1,2). To prevent a pulp exposure, indirect pulp treatment (IPT) and stepwise excavation have been advocated for deep carious lesions next to the pulp, but showing no signs or symptoms indicative of pulpal pathosis. These procedures include the incomplete removal of carious dentine to avoid a pulp exposure and the treatment of decay using a definitive restoration with biocompatible materials to stimulate healing and repair, and to protect against microleakage (1-5). IPT can be performed as a 1- visit treatment and stepwise excavation always involves an intermediate restoration before reentry (2).

Results of recent clinical studies suggest that a good marginal seal is more important than the type of lining material in achieving clinical success (4-6), as it is the key to preventings bacterial leakage and depleting any privates of nutrients for residual bacteria in remanent dentine. When dentine bacteria become isolated from the oral environment, their activity ceases and the pulp is able to recover (2-6).

Because restoration is an essential requirement for the long-term success of IPT, restorative materials must provide a permanent leak-proof seal. An adhesive restoration or preformed crowns are the most frequent restorations in IPT (1). However, in order to minimize the time spent by a child in the dental chair, simplify techniques and improve aesthetics results, researchers continue to explore alternative materials for primary teeth restoration.

Particularly in paediatric dentistry, resin-modified glass ionomer cements (resin-modified GICs) may act simultaneously as base and restorative material (7) given their ease of handling and mechanical advantages compared to conventional GICs, as well as their high success rates when used as IPT materials (6,8,9). Due to their biocompatibility, antibacterial capacity and good physical and mechanical properties, they provide good marginal sealing, little microleakage and a high retention rate (10,11). These advantages, coupled with the suitable performance as restorative materials in primary teeth (12), led them to be proposed as a dressing material and final restoration in IPT (6,7,9).

A recent clinical prospective study conducted by Kotsanos et al. (9) obtained a high rate of clinical success (96.5%) after a 32-months follow-up, using a single application (simultaneously, as base and final restoration) of resin-modified GIC for IPT in primary molars. These results suggest that resin-modified GICs helps isolate affected dentine from oral bacteria. Previous studies using different methods, however, have shown that leakage can occur to some degree with all resin-modified GICs (11,13). Antimicrobial agents used in the atraumatic restorative technique (ART) -a technique that also leave certain carious activity under restoration-before restoration with GIC, increase the survival of such restorations after two years of follow-up (14), suggesting that for IPT, the effect might be similar. Also, in the stepwise excavation technique, the use of antimicrobial agents before re-entry has led to a decreased number of bacteria in carious dentine arresting the caries process (for a review see Hayashi et al. 2011) (15).

Therefore, the purpose of this study was to evaluate, ex vivo, bacterial coronal leakage of different antimicrobial treatments, applied to the dentine in IPT.

## Material and Methods

In this study, we followed a protocol previously approved by the Ethics Committee of the University of Granada. Sixty non–carious teeth without cracks or any other enamel defects were collected. They were, single-rooted human mandibular premolars, with fully developed roots, and extracted for orthodontic reasons. The teeth were stored in 0.2% thymol solution at 4°C immediately after extraction. Any remaining tissue was mechanically removed. Using a standardized technique, digital radiographs were obtained from each tooth, placing them using a silicone mold as positioner. Once the image had been digitalized, two calibrated operators measured the total length of enamel and dentine to the pulp chamber, using DBSWIN® (Dürr Dental Medics Iberica, S.A., Madrid) program file and image processing. One trained operador prepared Class I occlusal cavities (3mm in diameter) with a round diamond bur (F.G. 801-029 Komet®) in a high speed handpiece under copious water spray, leaving 1 ± 0.2 mm of remanent thickness dentine (RTD) in all teeth, verified subsequently by another digital radiography.The crown length was then standardized by cutting, leaving a depth of 3mm in all cavities. The roots of all teeth were equalized to a length of 13 mm.

The teeth were randomly assigned into the five groups (n=10). Four groups received antimicrobial treatment using a microbrush with the following agents: 1% chlorhexidine (CHX) + 1% thymol varnish (Cervitec® Ivoclar, Vivadent AG), 2% CHX solution, 40% CHX varnish (EC40™ Biodent®, Certichem, Nijmenen, The Netherlands) and Clearfil™ Protect Bond (CPB, Kurakay Medical Inc.). The fifth group had no antimicrobial treatment. In all groups, the cavities were restored using Fuji II LC (GC Corp. Tokyo, Japan) according to the manufacturer´s recommendations. Although the clinical use of polyacrylic acid can enhance adhesion of GIC to the dentine, in our research it was not used so as to avoid interference with the antimicrobial agents tested.

Ten teeth served as controls: 5 positive (teeth with cavities and without antimicrobial treatment or restoration) and 5 negative (teeth with intact crowns).

-Microbiological leakage test

The external surface of each tooth, excepting 0.5 mm around the restoration, was covered with two layers of nail polish in order to prevent bacterial leakage. Negative controls were fully covered by two layers of varnish.

**Figure 1:**
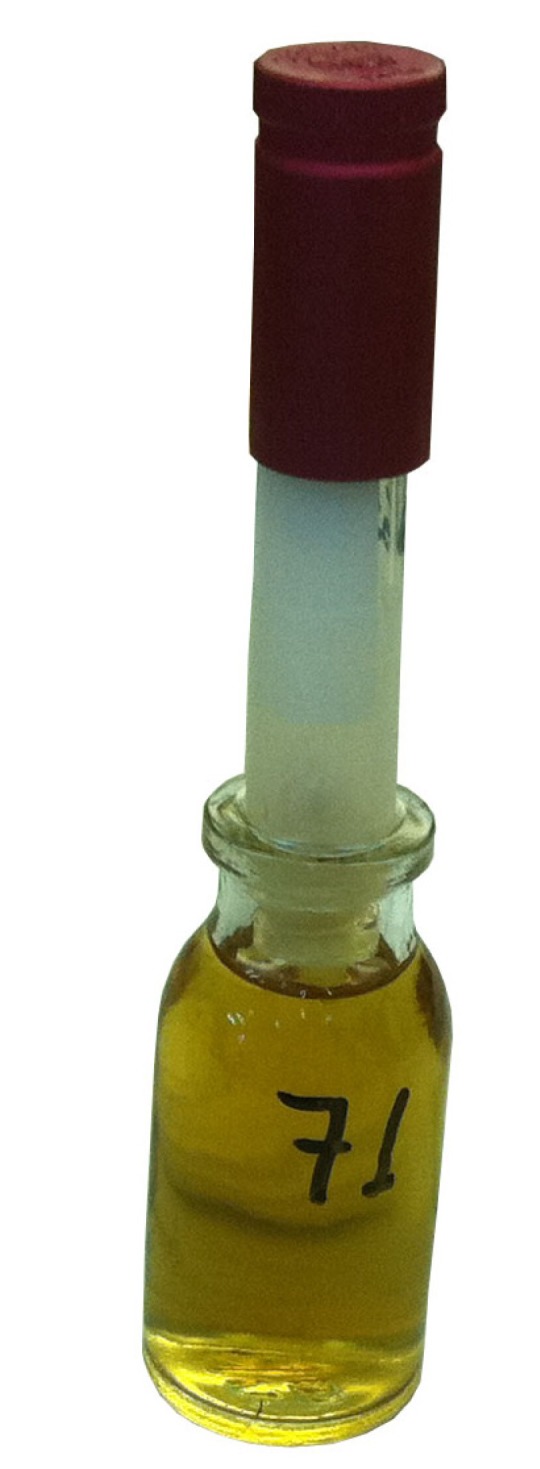
Two-chamber device for bacterial leakage.

**Figure 2:**
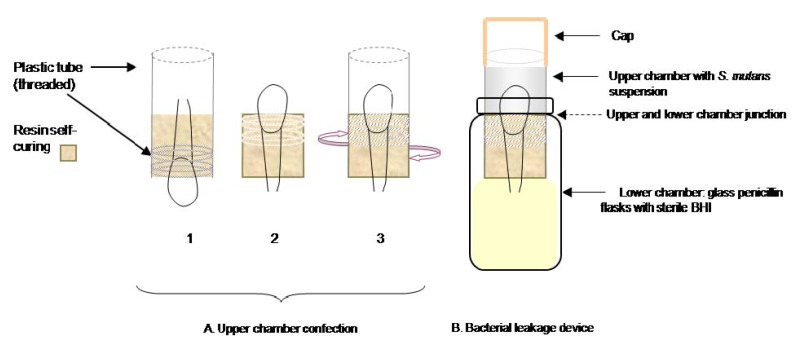
Schematic set-up of the two-chamber device used for bacterial leakage test. A. Upper chamber confection: 1) Specimens in self-curing acrylic resin 2) unmolded specimen and 3) screwed in a new tube. B. Bacterial leakage device.

The microbiological test consisted of a 2-chamber method described by Imura et al. (16) which we adapted for the dental crowns of our study (Fig. 1). For the upper chamber, all teeth were included in self-curing acrylic resin (Implex®, Dentsplay, Konstanz, Germany) using as molds, sections of plastic tubes, 10mm in diameter, (including the threaded part of the tube), leaving free the crown and 1.5 mm from the root. After completely setting, the specimens were unmolded and screwed into new tubes. Once in place the interface resin-tube was sealed using cyanoacrylate adhesive (Super Glue-3, Henkel Ibérica, S.A., Barcelona). The described device constituted the upper chambers, which were sterilized with ethylene oxide gas for 12 hours. Afterwards, the upper chambers were placed in lower chambers which consisted in glass penicillin flasks sterilized in an autoclave at 128º C and containing sterile Brain Heart Infusion broth (BHI, Scharlau Chemie S.A., Barcelona, Spain) until reaching the root end. The junctions between the two chambers were tightly sealed with Parafilm M™ (Pechiney Plastic Packaging, Chicago, IL, USA) and cyanoacrylate adhesive (Fig. 2).

The upper reservoirs were filled with 1 mL BHI broth containing 6 x 108 CFU/mL of Streptococcus mutans (SM) (ATCC 25175), maintaining the bacterial suspension in contact with the crown of the tooth. The devices were always handled in sterile conditions under a laminar flow hood (Nuaire, Plymouth, MN, USA) to avoid bacterial contamination. They were incubated at 37ºC. Five times a week, the medium in the upper chamber was replaced with freshly grown broth, and the viability and purity of the bacteria were checked every week.

The lower chambers of all devices were observed daily, turbidity time being recorded for each specimen as an indicator of leak-age. Once turbidity was present, a sample was inoculated in agar plates to check the presence of SM and the lack of contamination.

The proportion of unleaked samples over 45 days was calculated using the non-parametric Kaplan-Meier survival analysis. Differences among groups were tested using the log-rank test at a significance level of p<0.05. All statistical analyses were performed by means of SPSS 15.0 software (SPSS Inc, Chicago, IL, USA).

## Results

**Figure 3:**
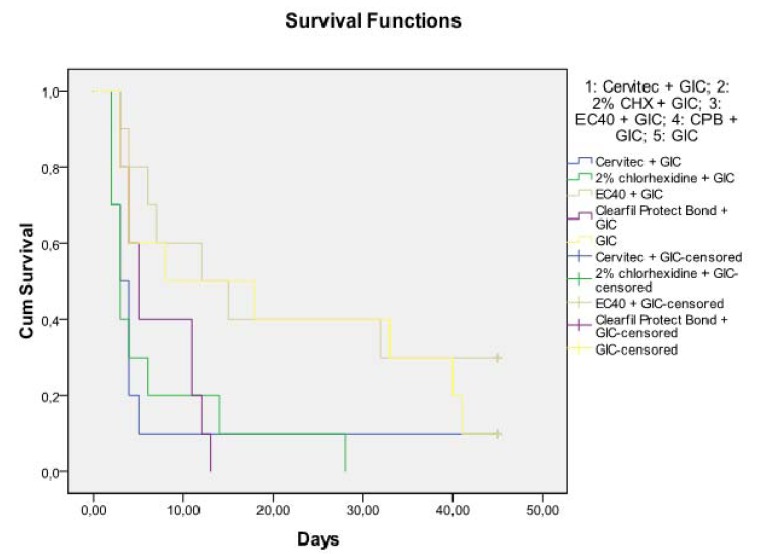
Kaplan–Meier survival probabilities (probability of being unleaked) for all groups. Censorated represents the proportion of the unleaked samples for each group at the end of the experimental period in the graph.

All positive controls exhibited bacterial leakage within 48 hours, whereas the lower chamber of negative controls remained uncontaminated throughout the experiment. The lower and the higher bound of leaked samples as well as the median are presented in ( Table 1). At 45 days of follow-up, all specimens of CPB and 2% CHX solution had leaked. The results of Kaplan-Meier survival analysis are shown in figure 3. Pair by pair comparison between protocols of survival analysis showed that the best results were obtained with EC40+GIC, followed by the group that recived no dentine antimicrobial treatment+GIC, without statistically significant differences between these two.

**Table 1:**
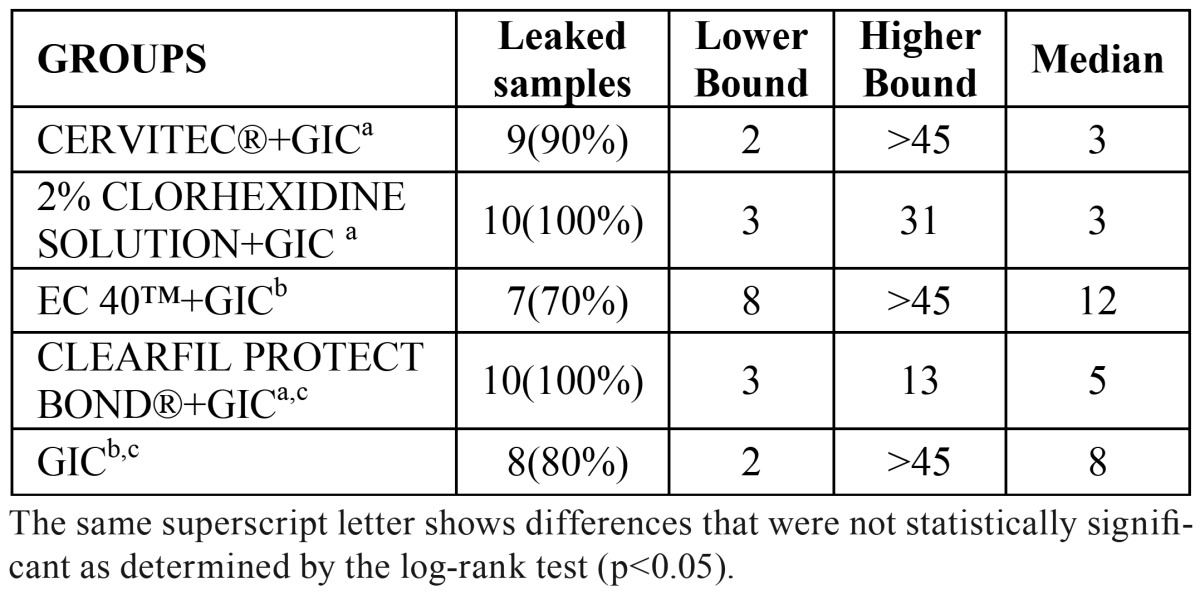
Number of leaked samples after 45 days (n=10 per group).

## Discussion

Diverse in vitro methods for evaluating the coronal leakage of different restorative materials have involved the use of dyes, scanning electron microscopy, fluid filtration techniques, electrochemical methods, radioisotopes, and bacteria (17,18). However, the use of bacteria as a marker is considered biologically and clinically more relevant (19).

The two-chamber model developed by Imura et al. (16) was adapted in our study for dental crowns. Although it is a static model that does not exactly simulate clinical conditions, it does not destroy the samples and by this method, it is also possible to study microleakage from the same specimen at intervals over extended periods (18,20). This method, mainly used for leakage studies in endodontics (16,18-20), has been applied successfully by Pameijer et al. (21) to assess marginal leakage in the cementation of crowns, although they inverted the chambers and used as bacteria Enterococcus faecalis. SM was the bacteria selected for our study, because it is the main organism causing dental caries (22). SM ATCC 25175 is a strain of reference widely used in antimicrobial susceptibility studies, and it exhibits a great capacity for invading tissues and forming biofilms (22,23).

The cavity preparation was similar in all groups, attempting to reproduce, as nearly as possible, the clinical of IPT. RTD is a clinically relevant parameter (8,24) and therefore it was strictly standardized (1 ± 0.2 mm), because the pulp´s repair capacity is excellent when the carious lesion remains 1 mm or more away from the pulp (25). When IPT is carried out in vivo, the production of reactive dentine by odontoblast is also influenced by RTD (26).

None of the protocols used in our study could completely prevent microleakage of SM. Though the lowest number of leaked samples was in the group EC40+GIC, this result was not significantly different than the GIC group without treatment. Lower survival values of leakage, but statistically similar between them, were obtained in the groups CPB+GIC, Cervitec®+GIC and 2% CHX solution+GIC.

The results of the present study can be tied to different factors. On the one hand, the antibacterial effect of CHX is concentration-dependent (27) and its antimicrobial substantivity also depends on the number of CHX molecules available to interact with the dentine (27). Although microleakage occured in the EC40™ group, it was considerably delayed in time, probably due to the high antimicrobial effectiveness deriving from the great concentration of active agent in EC40™. Also, the vehicle of CHX used could have influenced these results. EC40™ varnish has a consistency similar to a gel. Although the cavity was dried, some material could have been trapped in the dentine, acting as a slow release device (28). The lower survival obtained by the other varnish, Cervitec®, could be due to its very low CHX concentration (1%) despite also having 1% thymol. Accordingly, EC40™ has been reported to exert a longer effect in reducing mutans streptococci salivary levels than 1% CHX varnish (29).

Before using a Resin-modified GIC, the use of polyacrylic acid is normally recommended, given its capacity to condition the dentine, removing the smear layer and partly demineralizing the dentine. This facilitates the infiltration of the HEMA contained in the resin-modified GIC into the exposed collagen network of the dentine (30). In our methodology, however, polyacrylic acid was not applied prior to antimicrobial treatment, because the presence of a specific bond between the chlorhexidine imine groups and polyacrylic acid carboxylic groups has been confirmed (31). This could explain the reduced antimicrobial effect when the two agents are combined (32). Although no in vivo studies to date have demonstrated this antagonism, we believe that such a negative interaction is very likely, and underline this aspect as deserving further study.

The survival of CPB+GIC group showed an intermediate value, although statistically minor than GIC without treatment. The antibacterial effect of CPB is related to the antibacterial molecule MDPB (33) which is not completelly immobilized after the curing of the adhesive (34). However, this advantage was not able to reduce bacterial leakage; and moreover these comparatively poor results suggest that the use of this dentine adhesive could act as a barrier impeding the adherence of GIC to the dentine. This property inherent to GIC (10) could explain the optimal results (similar to EC40™) when it was used as a sealing material without any type of previous dentine treatment.

The limited survival of specimens treated with 2% CHX solution, is somewhat surprising. A priori we surmised that CHX in solution could improve the result obtained in the GIC group without treatment. Upon drying the cavity, any excess solution would be eliminated, but a monolayer of active CHX molecules would remain adhered to the underlying dentine. However, the fast bacterial leakage of all samples appears to indicate that the positive antimicrobial effect of CHX may have been neutralized by a potential interference in the GIC-dentine adhesion. It was recently shown that a 2% CHX solution decreases adhesion of resin-modified GIC (Fuji II LC) to bovine dentine (35). Further investigations are needed to determine if the incorporation of additional agents into GIC can improve its antimicrobial effect without interfering with its mechanical properties.

In conclusion, the use of GIC-without pretreatment of the dentine, as only material in IPT, led to a delay of coronal leakage by SM without statistically significant differences when compared with the group treated with 40% CHX varnish.

## References

[1] Rodd HD, Waterhouse PJ, Fuks AB, Fayle SA, Moffat MA (2006). UK National Clinical Guidelines in Paediatric Dentistry: Pulp therapy for primary molars. Int J Paediatr Dent.

[2] American Academy of Paediatric Denstistry (2010). Guideline on pulp therapy for primary and young permanent teeth. Reference Manual 2010-11. Pediatr Dent.

[3] Fuks AB (2008). Vital pulp therapy with new materials for primary teeth: new directions and treatment perspectives. Pediatr Dent.

[4] Büyükgüral B, Cehreli ZC (2008). Effect of different adhesive protocols vs calcium hydroxide on primary tooth pulp with different remaining dentin thicknesses: 24 month results. Clin Oral Investig.

[5] Falster CA, Araujo FB, Straffon LH, Nör JE (2002). Indirect pulp treatment: in vivo outcomes of an adhesive resin system vs calcium hydroxide for protection of the dentin-pulp complex. Pediatr Dent.

[6] Marchi JJ, de Araujo FB, Froner AM, Straffon LH, Nör JE (2006). Indirect pulp capping in the primary dentition: a 4 year follow-up study. J Clin Pediatr Dent.

[7] Marchi JJ, Froner AM, Alves HL, Bergmann CP, Araújo FB (2008 ). Analysis of primary tooth dentin after indirect pulp capping. J Dent Child (Chic).

[8] Gruythuysen RJ, van Strijp AJ, Wu MK (2010). Long-term survival of indirect pulp treatment performed in primary and permanent teeth with clinically diagnosed deep carious lesions. J Endod.

[9] Kotsanos N, Arizos S (2011 ). Evaluation of a resin modified glass ionomer serving both as indirect pulp therapy and as restorative material for primary molars. Eur Arch Paediatr Dent.

[10] Sidhu SK (2010). Clinical evaluations of resin-modified glass-ionomer restorations. Dent Mater.

[11] Abd-El-Halim S, Zaki D (2011 ). Comparative evaluation of microleakage among three different glass ionomer types. Oper Dent.

[12] Qvist V, Poulsen A, Teglers PT, Mjör IA (2010 ). The longevity of different restorations in primary teeth. Int J Paediatr Dent.

[13] Wadenya R, Smith J, Mante F (2010). Microleakage of nano-particle-filled resin-modified glass ionomer using atraumatic restorative technique in primary molars. N Y State Dent J.

[14] Ersin NK, Aykut A, Candan U, Oncag O, Eronat C, Kose T (2008). The effect of a chlorhexidine containing cavity desinfectant on the clinical perfomance of high-viscosity glass–ionomer cement following ATR: 24-month result. Am J Dent.

[15] Hayashi M, Fujitani M, Yamaki C, Momoi Y (2011). Ways of enhancing pulp preservation by stepwise excavation- A systematic rewiew. J Dent.

[16] Imura N, Otani SM, Campos MJA, Jardim EG, Zuolo ML (1997). Bacterial penetration through temporary restorative materials in root canal treated teeth in vitro. Int Endod J.

[17] Raskin A, D’Horre W, Gonthier S, Degrange M, Déjou J (2001). Reliability of in vitro microleakage tests: a literature review. J Adhes Dent.

[18] Veríssimo DM, do Vale MS (2006). Methodologies for assessment of apical and coronal leakage of endodontic filling materials: a critical review. J Oral Sci.

[19] Timpawat S, Amornchat A, Trisuwan W, Dip G (2001). Bacterial Coronal Leakage after obturation with Three Root Canal Sealers. J Endod.

[20] Celik EU, Yapar AG, Ateş M, Sen BH (2006). Bacterial microleakage of barrier materials in obturated root canals. J Endod.

[21] Pameijer CH, Zmener O, Alvarez-Serrano S, Garcia-Godoy F (2010). Sealing properties of a calcium aluminate luting agent. Am J Dent.

[22] Loesche WJ (1986). Role of Streptococcus mutans in human dental decay. Microbiol Rev.

[23] Aas JA, Griffen AL, Dardis SR, Lee AM, Olsen I, Dewhirst FE (2008). Bacteria of dental caries in primary and permanent teeth in children and young adults. J Clin Microbiol.

[24] Murray PE, Smith AJ, Garcia-Godoy F, Lumley PJ (2008 ). Comparison of operative procedure variables on pulpal viability in an ex vivo model. Int Endod J.

[25] Reeves R, Stanley HR (1966). The relationship of bacterial penetration and pulpal pathosis in carious teeth. Oral Surg Oral Med Oral Pathol.

[26] Murray PE, Smith AJ, Windsor LJ, Mjör IA (2003). Remaining dentine thickness and human pulp responses. Int Endod J.

[27] Mohammadi Z, Abbott PV (2009). The properties and applications of chlorhexidine in endodontics. Int Endod J.

[28] Wetman S (2004). Antimicrobials in future caries control?. A review with special reference to chlorhexidine treatment. Caries Res.

[29] Ribeiro LG, Hashizume LN, Maltz M (2007). The effect of different formulations of chlorhexidine in reducing levels of mutans streptococci in the oral cavity: A systematic review of the literature. J Dent.

[30] Tanumiharja M, Burrow MF, Tyas MJ (2000). Microtensile bond strengths of glass ionomer (polyalkenoate) cements to dentine using four conditioners. J Dent.

[31] Musial W, Kokol V, Voncina B (2009). The preliminary assessment of chlorhexidine and lidocaine release from preparations of anionic polymer, evaluated by the conductivity measurements. Polim Med.

[32] Botelho MG (2005). The antimicrobial activity of a dentin conditioner combined with antibacterial agents. Oper Dent.

[33] Imazato S, Torii Y, Takatsuka T, Inoue K, Ebi N, Ebisu S (2001). Bactericidal effect of dentin primer containing antibacterial monomer methacryloyloxydodecylpyridinium bromide (MDPB) against bacteria in human carious dentin. J Oral Rehabil.

[34] Feuerstein O, Matalon S, Slutzky H, Weiss E (2007). Antibacterial properties of self-etching dental adhesive systems. JADA.

[35] Wangpermtam P, Botelho MG, Dyson JE (2011). Effect of contamination and decontamination on adhesion of resin-modified glass ionomer cement to bovine dentin. J Adhes Dent.

